# The relation between bystanders’ behavioral reactivity to distress and later helping behavior during a violent conflict in virtual reality

**DOI:** 10.1371/journal.pone.0196074

**Published:** 2018-04-19

**Authors:** Ruud Hortensius, Solène Neyret, Mel Slater, Beatrice de Gelder

**Affiliations:** 1 Institute of Neuroscience and Psychology, School of Psychology, University of Glasgow, Glasgow, Scotland, United Kingdom; 2 Brain and Emotion Laboratory, Department of Cognitive Neuroscience, Faculty of Psychology and Neuroscience, Maastricht University, EV Maastricht, The Netherlands; 3 Experimental Virtual Environments for Neuroscience and Technology (EVENT) Laboratory, Department of Clinical Psychology and Psychobiology, University of Barcelona, Barcelona, Spain; 4 Institució Catalana de Recerca i Estudis Avançats (ICREA), Barcelona, Spain; 5 Department of Computer Science, University College London, London, England, United Kingdom; 6 Department of Psychiatry and Mental Health, University of Cape Town, J-Block, Groote Schuur Hospital, Observatory, Cape Town, South Africa; University of Bologna, ITALY

## Abstract

The occurrence of helping behavior is thought to be automatically triggered by reflexive reactions and promoted by intuitive decisions. Here, we studied whether reflexive reactions to an emergency situation are associated with later helping behavior in a different situation, a violent conflict. First, 29 male supporters of F.C. Barcelona performed a cued-reaction time task with a low and high cognitive load manipulation, to tap into reflexive and reflective processes respectively, during the observation of an emergency. Next, participants entered a bar in Virtual Reality and had a conversation with a virtual fellow supporter. During this conversation, a virtual Real Madrid supporter entered and started an aggressive argument with the fellow supporter that escalated into a physical fight. Verbal and physical interventions of the participant served as measures of helping behavior. Results showed that faster responses to an emergency situation during low, but not during high cognitive load, were associated with more interventions during the violent conflict. However, a tendency to describe the decision to act during the violent conflict as intuitive and reflex-like was related to more interventions. Further analyses revealed that a disposition to experience sympathy, other-oriented feelings during distressful situations, was related to self-reported intuitive decision-making, a reduced distance to the perpetrator, and higher in the intervening participants. Taken together, these results shed new light on helping behavior and are consistent with the notion of a motivational system in which the act of helping is dependent on a complex interplay between intuitive, reflexive and deliberate, reflective processes.

## Introduction

Functional altruism and socially motivated helping, behaviors that benefits the recipient but with a cost to the actor, are observed throughout the animal kingdom [1]. Humans as young as 12 to 14 months provide help [2–4]. Chimpanzees demonstrate costly helping in a variety of situations with and without reward [2,3,5,6]. There is also considerable evidence that rats exhibit helping behavior [7–12] and there is even evidence of functional altruism in ants [13–15]. The debate is ongoing whether all of these costly behaviors can be interpreted as a form of empathy [16,17], but the crucial point is the occurrence of helping behavior. The fact that helping behavior is so widespread suggests the presence, at least at some level, of a phylogenetically ancient mechanism that gives rise to the variety of prosocial and empathic behaviors in humans [18,19]. Indeed, a recent theoretical model highlights offspring care [20] as a possible hard-wired, evolutionarily conserved mechanism that provides the foundation for helping behavior and other functional altruistic behaviors. Importantly, as the species that show helping behavior differ greatly in cognitive capacities, it is unlikely that these capacities play a crucial role in the preparation and execution of helping behavior. Thus, the occurrence of helping behavior is likely to be relatively independent of cognitive abilities and to rely more on automatically triggered fixed-action patterns [20,21].

Is helping behavior a reflexive action? Statements by people that provided help under extreme circumstances are rated as automatic and reflex-like, rather than deliberate or reflective [22]. Studies directly manipulating decision time found that under time pressure people are more cooperative as a result of a more intuitive decision-making process ([23–26], but see [27,28]). Time pressure also increased reported inclination of individuals to sacrifice a preferred activity to help their romantic partner or best friend [29]. Priming individuals with an intuitive compared to reflective cognitive state resulted in increased contribution to the common good [30]. Similarly, increased cognitive load resulted in more generous offers to others [31,32]. Taken together, intuition compared to deliberation is related to increased prosocial behavior. But are inter-individual differences in reflexive- and reflective-like reactions, or the extent to which people rely on intuition or deliberation, associated with later prosocial behavior?

Here, we address this question by investigating whether reflexive and reflective behavioral responses to one situation (an emergency) are related to helping behavior in a different situation (a violent conflict). The processing of and the reaction to distress is a likely predictor of helping behavior [33]. For example, Marsh, Kozak and Ambady [34] found across a series of experiments that the ability to recognize facial expressions of fear, a clear signal of distress in another individual, was related to greater prosocial behavior. In the present study, covert behavioral reactivity to distress was measured by reaction times of the participant when observing an emergency situation in which a woman is in need of help. This behavioral measurement was followed by a Virtual Reality (VR) procedure that measured helping behavior during a violent conflict. While previous studies have used situations and measures of helping and other prosocial behaviors that are relevant to the individual, for example sharing in an economic game, they are low in terms of risk, in danger to the participant and unlikely to be encountered in daily life. One way to circumvent this is to use the powerful tool of VR. This allows researchers to explore situations that cannot be created in reality because they are either impractical, unethical or too dangerous for the participant. It further supports ecological validity while simultaneously maintaining experimental control, and measuring genuine phenomenological, behavioral and physiological outcomes [35–38]. In the present study, participants were confronted with a violent conflict between two individuals in a virtual bar [39]. The number of physical and verbal interventions made by the participant during this conflict served as helping behavior measures. This procedure has successfully been used to study helping behavior in onlookers during this violent incident in which a victim is verbally and physically attacked by a perpetrator [39].

We tested the hypothesis as to whether reaction times to an emergency situation during high cognitive load, indicative of reflexive, automatic responses, but not during low cognitive load, indicative of reflective, deliberate responses, would be related to helping behavior during a later violent conflict. We further expected that a tendency by the participant to describe the decision-making process during the violent conflict as intuitive, fast and reflexive would be positively related to the number of interventions. Lastly, several ideas on the role of dispositional sympathy arose as a result of the outcome of the experiment that required additional analyses not anticipated at the start.

## Materials and methods

### Participants

Participants interested in football (soccer) were recruited by advertisements around the University of Barcelona campus and by word-of-mouth. The sample size (*n* = 30) was determined before the start of the study, based on previous literature [34,39,40]. Potential participants were required to complete an online questionnaire that asked about their interest in football and their favorite team and level of support for this team. Twenty-nine male supporters of F.C. Barcelona, between 18 and 29 years of age, were eventually recruited. One additional participant was excluded before the start of the study because of technical difficulties. The median level of support for F.C. Barcelona on a scale from 1 (not at all) to 7 (very much so) was 5 with an interquartile range of 2. **Table A in S1 File** reports additional sample characteristics. Participants had normal or corrected-to-normal vision and were screened for contra-indications for VR (history of epilepsy, recent psychotropic drug intake). Participants received oral and written information prior to the study, but remained naïve to the goal of the experiment, and provided written informed consent. The compensation was ten Euros. The study was approved by the Comissió Bioètica of Universitat de Barcelona (IRB00003099) and carried out in accordance with the standards set by the Declaration of Helsinki.

### Cued reaction time task

**Fig 1:**
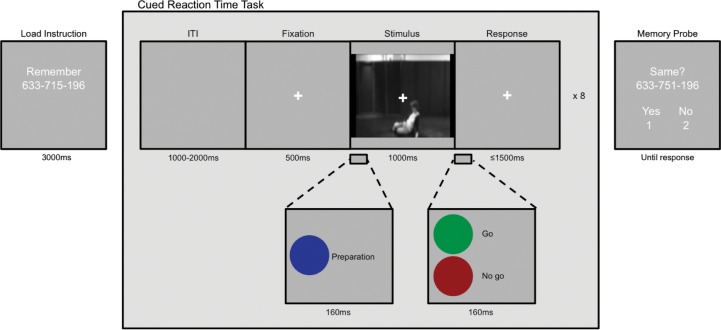
Cued reaction time task with cognitive load manipulation. Before each reaction time task block a low or high load instruction screen was presented. Participants were instructed to respond as fast as possible to the go cue with the index finger of their dominant hand while simultaneously remembering the phone number. Between a preparation and a response cue, a video clip depicting an emergency or nonemergency situation was shown. After eight reaction time trials a memory probe was shown and participants indicated if the phone number was the same as the to be remember one.

An adapted version of the cued reaction time task with cognitive load manipulation from Hortensius, Schutter and de Gelder [40] was used (**Fig 1**). In this task, a preparation cue (blue dot) is presented before a response cue. Participants are instructed to respond as fast as possible to a go cue (green dot), but to withhold their response to a no go cue (red dot). Reaction times in the cued reaction time task serve as the main outcome measures and have previously been used to index action preparation or readiness [40]. Participants are able to prepare their response to the response cue after the presentation of a preparation cue [41,42]. Similar to other social cognitive tasks such as emotional Stroop, go/no-go and gaze-cueing tasks, responses in the cued reaction time task are influenced by the stimuli presented between the preparation and response cue and vary between individuals [43,44]. Faster reaction times are associated with increased action preparedness, while slower reaction times indicate decreased action preparedness with respect to the presented situation. The use of a cognitive load task manipulation is a well-established method to impose restrictions on cognition and assess the role of intuition and automaticity [31,32,45,46]. Importantly, it does not rely on problematic reaction time reverse inference, i.e. faster responses are reflexive or intuitive and slower responses are reflection or deliberate [47]. During a cognitive load manipulation, participants are asked to perform a secondary task (e.g., memorizing a number) that varies in difficulty. During a low-load condition, when the secondary task is easy, the cognitive system is accessible and can influence ongoing behavioral processes, while in a high-load condition the secondary task is more difficult leaving the cognitive system engaged and relatively inaccessible. If a behavioral process of interest is intact during the high-load condition, it can be inferred that this process is relatively independent from cognition and can be referred to as reflexive and automatic, opposed to reflective and deliberate.

Reaction times were measured by response button presses made with the index finger of the dominant hand of the participant. All cues are presented for 160ms, and 25% of the trials were no go trials. In between the preparation and response cue, a 1s video clip depicting an emergency (falling woman) or non-emergency situation (woman standing up) was presented [48]. During the reaction time task participants were instructed to simultaneously remember a phone number (see [46]). This phone number could be easy (e.g, 888–888–888, low cognitive load) or hard to memorize (e.g., 643–687–237, high cognitive load). Before the onset of an eight trial reaction time block, a load instruction screen was presented for 3000ms. At the end of the block, participants indicated whether the presented phone number was the same as the to be remembered number. A pilot experiment (*n* = 5) revealed that manipulation of cognitive load was successful. Accuracy was higher in the low cognitive load condition, 90% correct, compared to the high cognitive load condition, 67.50% correct, with a mean difference between condition of 22.50 [9.51, 35.49] (throughout the article, we report the lower and upper bounds of the 95% confidence interval within square brackets).

After practice of the cued reaction time task (three trials, one no go trial), the cognitive load manipulation was added and participants completed a low and high cognitive load block each containing three reaction time trials (one no go trials). For the practice trials video clips of a woman standing and waiting were used. Participants completed 128 experimental trials (4 conditions * 32 trials).

### Virtual reality scenario

**Fig 2:**
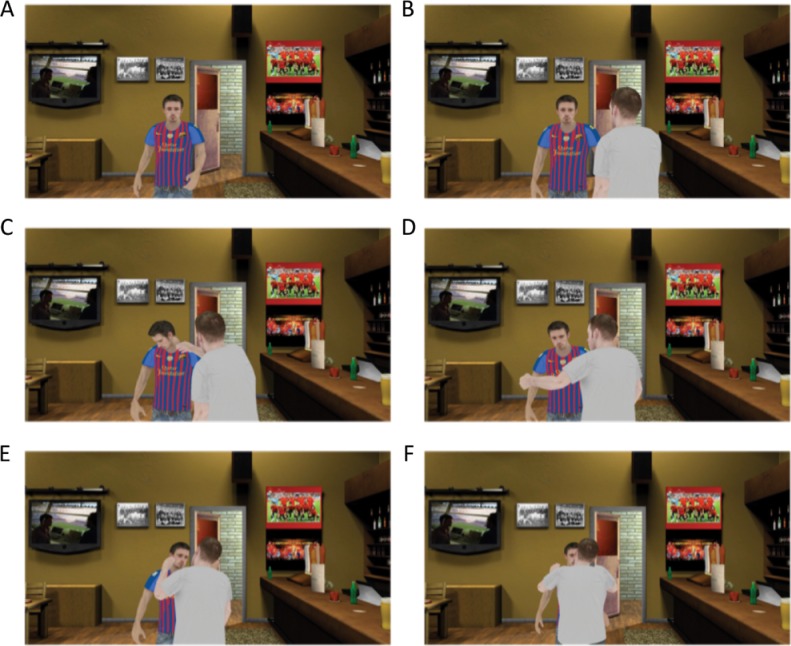
Visual representation of the scenario. While the participants had a conversation with the victim (A), the perpetrator walks over to victim (B) and starts an argument (C—E) that becomes increasingly intense and eventually escalated into a physical attack (F). Please note that the viewpoint of these frames do not match the viewpoint of the participants as the actual scenario was in three-dimensional stereo vision and based upon the position of the participants in the CAVE.

An adapted scenario from Slater and colleagues [39] was used. In the scenario the participants had a short free-flow conversation about F.C. Barcelona (e.g., results, favorite player) with a male virtual human (V, victim), a fellow Barça supporter who was wearing a Barça shirt. While the utterances of V had been prerecorded, the selection of his responses was made by an experimenter, based on the response of the participants, allowing for what seemed to be a normal conversation (mean ± SD duration: 103 ± 24s). During the conversation, another male virtual human (P, perpetrator), wearing a Real Madrid shirt, entered and sat at the bar. After a few minutes he stood up and walked towards V starting an argument about V’s shirt and support for Barça. During the argument V took a submissive, conciliatory role, and occasionally made eye contact with the participant. Over time the verbal attack of P on V became increasingly intense and escalated into a physical attack of P on V. The conflict between P and V was the same for all participants (total time of conflict: 135s). **Fig 2** provides a visual representation of the scenario.

### Virtual reality system

The program was developed in the XVR programming platform [49], with the virtual characters animated with HALCA software [50], and delivered by a ‘CAVE’ system [51]. The CAVE consists of three back-projected walls and a front-projected floor (1920 × 1200 pixels resolution), each measuring 3.80 by 2.25m, using Christie Digital Mirage WU3 three-chip digital light processing projectors driven by a Master-node with four slave-nodes. Alternate images at 60Hz were presented to each eye of the participants synchronized with the projectors using RealD 3D CrystalEyes stereo shutter glasses resulting in overall 3D stereo vision. A head tracker (InterSense IS900) was mounted on top of the glasses and fed the participants’ head position and movement into XVR to allow constant updating of the virtual environment based on the position and movement of the participant. Participants were fitted with headphones for auditory stimuli, and a microphone for voice recordings. The entire scenario was recorded using a video camera from the top of the CAVE filming the participant from the back to preserve anonymity.

### Decision-making questionnaire

To assess self-reported intuitiveness versus deliberativeness of the decision-making process during the conflict we included a questionnaire based on Rand and Epstein [22]. The questionnaire assessed the intuitiveness of the decision to verbally or physically intervene or not at two time points during the conflict as well as the overall decision to act (five items). After the participants were made familiar with the definition of intuitive and reasoned decisions following Rand and Epstein [22], they rated the statements on a scale from 1 (intuitive/fast) to 5 (reasoned/slow). The intervention scale of the decision-making questionnaire had a high reliability, Cronbach’s α = .82. **Table B in S1 File** reports the items and responses. General intuitiveness was also assessed by non-intervention related aspects. These three items, assessing the intuitiveness of the interaction with V and internal reactions during the conflict, had a low reliability, Cronbach’s α = .28, and were discarded from further analyses.

### Trait empathy questionnaire

Trait levels of cognitive and affective components of empathy were measured with the Interpersonal Reactivity Index [52–54]. Perspective taking (the capacity to understand the thoughts and feelings of another individual) and fantasy (the ability to transpose oneself to a fictional situation) measure the cognitive component. The affective component is made up of the personal distress and empathic concern subscales. These two subscales differ in terms of focus of the emotional reaction. The former measures the experience of discomfort in the observer in response to distress in others (a self-oriented emotional reaction), while the latter measures sympathy and compassion in the observer for less fortunate others (an other-oriented emotional reaction). To prevent confusion with the general concept of empathy, we use the term sympathy when referring to the trait measure of the other-oriented emotional reaction of empathic concern. The four scales (**Table C in S1 File)** all had high reliability in the current sample, Cronbach’s α ≥ .72.

### Presence questionnaire

Presence of the participant in the virtual word was assessed using a previous developed questionnaire [55–57]. Presence is the notion that an individual feels and behaves as if he is in the virtual world despite knowledge of the virtual aspect. On a scale from 1 (low presence) to 7 (high presence) participants answered several questions that assess both the place illusion (the sensation of being in the virtual bar) and plausibility (the illusion that the conversation and conflict occurring in the bar were real). Both the place illusion and plausibility scales have good internal reliability in the present study, Cronbach’s α = .85 and α = .87 respectively.

### Interview

As in Slater et al. [39] a short interview was conducted to asses phenomenological responses during the Virtual Reality scenario. The participants were asked to describe their feelings and responses during the violent conflict, how realistic these responses were, and what aspects might have increased the possibility of intervening. Lastly, participants were asked to describe aspects that made them feel outside of the scenario.

### Procedure

After explanation of the procedures by the experimenter, the participant provided informed consent and answered several questions on the intake of psycho-active drugs and alcohol, frequency of video game playing, level of expertise in informatics and programming, and past experience with virtual reality. The study consisted of three parts; 1) cued reaction time task, 2) virtual reality scenario, and 3) questionnaires and interview. After completion of the cued reaction time task, the VR procedure began and participants were told that they would enter a bar and meet some people inside with whom they were free to interact. Before entering the virtual environment, participants were fitted with the 3D glasses, head tracker, headphones, and a microphone. Participants entered the virtual bar and were asked to describe the environment in detail. Following completion of this familiarization period, the VR scenario started. The program was terminated after the physical attack, and participants exited the virtual environment. The session was concluded with completion of the questionnaires and interview. After debriefing the participants received their payment.

### Data processing

#### Reaction time analysis

Reaction times below <150 ms and >1500 ms (responses after the offset of the response screen), as well as incorrect trials were removed from analysis (mean ± SD percentage of trials removed: 1.86 ± 1.55%). We calculated the bias score for both the low and high cognitive load condition separately by subtracting the reaction times in the nonemergency from the emergency situation. Negative values indicate faster responses to the emergency situation. For the main analysis we corrected for general task effects of the cognitive load manipulation by removing the variance explained by the overall task performance during the cued reaction time task. A linear regression was used for each bias score with accuracy low–high cognitive load as a predictor [40], allowing us to assess the unique contributions of each condition by using the standardized residual of each of the bias scores. Given the a priori predictions we used Spearman correlations to test if the emergency–nonemergency bias scores (standardized residual) during the cued reaction time task were correlated with the number of intervention during the conflict in VR.

#### Video coding

Helping behavior was defined as the number of verbal and physical interventions of the participant during the conflict in the virtual bar. Two people independently coded the videos. One of the experimenters (S.N.) and one independent person were instructed to count the number of verbal and physical interventions. The same definition of interventions was used as in Slater and colleagues [39]. Utterances directed at P or V were counted as verbal interventions. Laughing or sighs were not counted as interventions. Physical interventions were defined as either an action together with a verbal intervention, or an action directed at P or V (e.g., stepping in-between P and V or a hand movement to signal P to stop). The number of counted interventions was highly correlated between the two coders; verbal interventions *r*_s_ = .89, *p* < .001, physical interventions, *r*_s_ = .95, *p* < .001. The coding of the videos was carefully compared between the two coders and a final review of all the videos was performed to provide solutions for discrepancies and to make sure that no intervention was missed. This revealed that the slightly lower correlation for the verbal interventions was because one of the coders did not count the whistles of a participant as verbal interventions. These whistles were used by the participant to get the attention of P and were counted as verbal interventions after the final review.

#### Tracking

Throughout the VR scenario the head orientation and position of the participant as well as the position of V and P were tracked and recorded (X/Y/Z-coordinates). Here X is left/right, Y is up/down, and Z is forward/backward. The unit is meters and the origin (0, 0, 0) of the CAVE lies on the front wall center floor. The signal was offline downsampled to 60Hz using Spline Interpolation (with a pre-downsample filter of 27Hz, 24dB/oct). When tracking was lost, the data during that time window was excluded (four participants, with time windows of ~2, 4, 9, and 16.5s). Besides mean and standard deviation displacement in terms of X- and Z-coordinates, the following outcome measures were calculated. Distance to V was calculated with the following formula:

$$\sqrt{{\left(x_{i}-x_{j}\right)}^{2}+{\left(z_{i}-z_{j}\right)}^{2}}$$

Where *x_i_* and *z_i_* are the coordinates for V and *x_j_* and *z_j_* are the coordinates for the participant. The mean and standard deviation distance to V were calculated separately for the conversation and conflict period. The same formula, but limited to the conflict period, was used to calculate the distance to P. Next, we calculated the time spent in proximity of V and P. Using the definition of social distances from proxemics [58], which are also used in virtual reality studies [59–61], we calculated the time spent in public (between 3.7 m and 7.6m), social (between 1.2 m and 3.7 m), personal (between 0.46 and 1.2 m), and intimate (<0.46 m) distances. The well-known personal space bubble corresponds to an interpersonal distance of around 40 cm.

## Results

### Preliminary analyses

The cognitive load manipulation was successful, accuracy was higher in the low cognitive load condition, mean percentage correct: 98.28 [96.61, 99.94], compared to the high cognitive load condition, 72.41 [65.07, 79.76], *t*(28) = 7.62, *p* < .001, d = 1.42 [0.89, 1.93], mean difference = 25.86 [18.91, 32.81]. **Table D in S1 File** reports the reaction times and bias scores for the cued reaction time task. Participants were faster in responding to emergency and nonemergency situations alike in the low cognitive load condition, mean in ms: 318.08 [302.44, 333.72], compared to the high cognitive load condition, 330.78 [313.16, 348.40]. Besides a main effect of cognitive load, *F*(1,28) = 9.59, *p* = .004, η^2^ = 0.26, no main effect for situation or interaction between situation and cognitive load was found, *F*(1, 28) = 0.00, p = .983 and *F*(1, 28) = 0.26, *p* = .615 respectively. Bias scores did not differ between the two cognitive load conditions, *t*(28) = 0.51, *p* = .615.

Participants did not voice any potential relation between parts of the study (e.g. cued reaction time task and virtual reality) and no side effects to the virtual reality were reported. Median response (and interquartile range) for the place illusion scale was 4.25 (2.63) and for the plausibility scale 3.67 (2.17). **Tables E**-**F in S1 File** report the rating for the individual items. The interview showed that the scenario was successful in eliciting realistic responses and feelings such as anger, sympathy, distress, and helplessness. The responses to the interview questions (**Tables G-J in S1 File**) are consistent with the findings of Slater el al. [39].

The mean number of interventions was 9.07 [4.84, 13.30], with 3.38 [1.64, 5.12] physical interventions, and 5.69 [3.08, 8.30] verbal interventions. From the 29 participants, 9 refrained from any intervention. The first intervention was 26.20 [13.52, 38,88] s after onset of the conflict. As the number of verbal and physical interventions were significantly correlated, *r*_s_(29) = .83, *p* < .001, we combined them in one measure of helping behavior.

**Fig 3:**
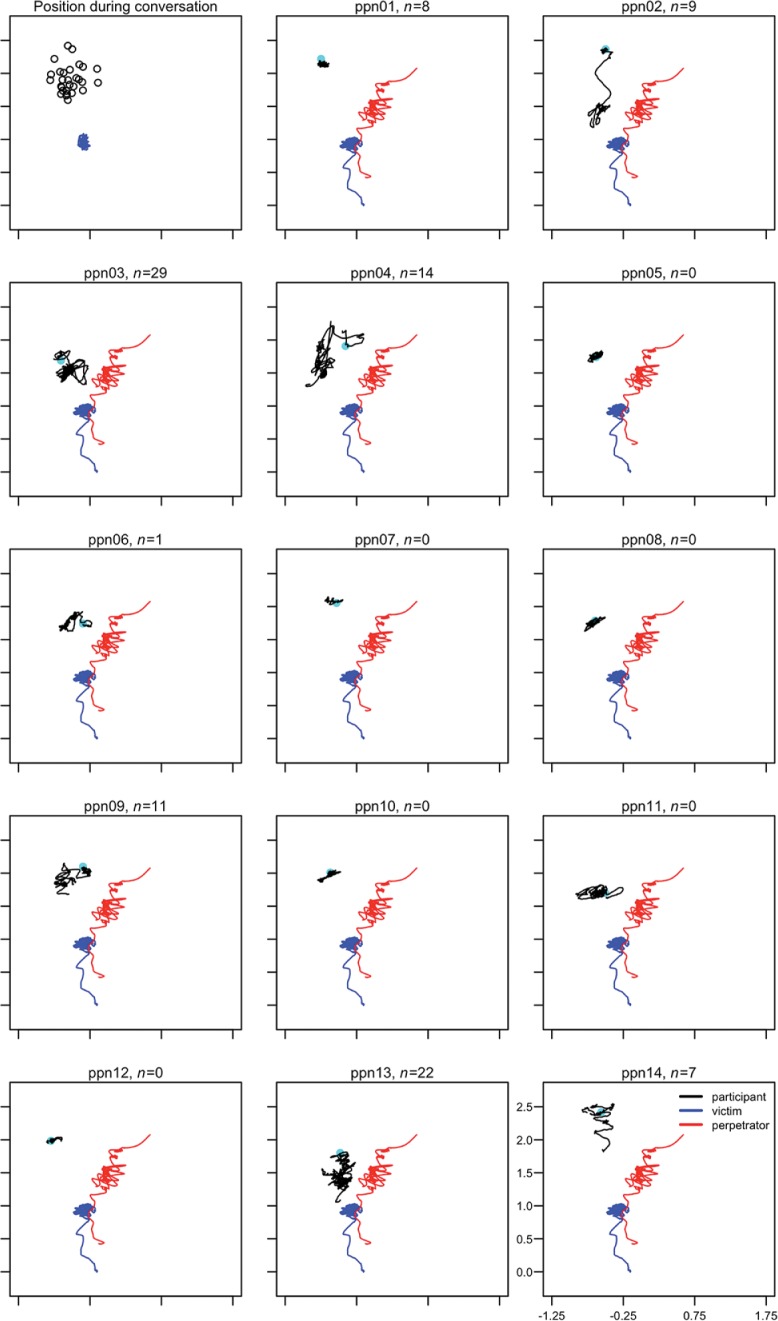
Movement of participants 1–14 and V and P during the violent conflict. *n* indicates number of interventions, cyan dots indicate the position of the participant during the conversation.

**Fig 4:**
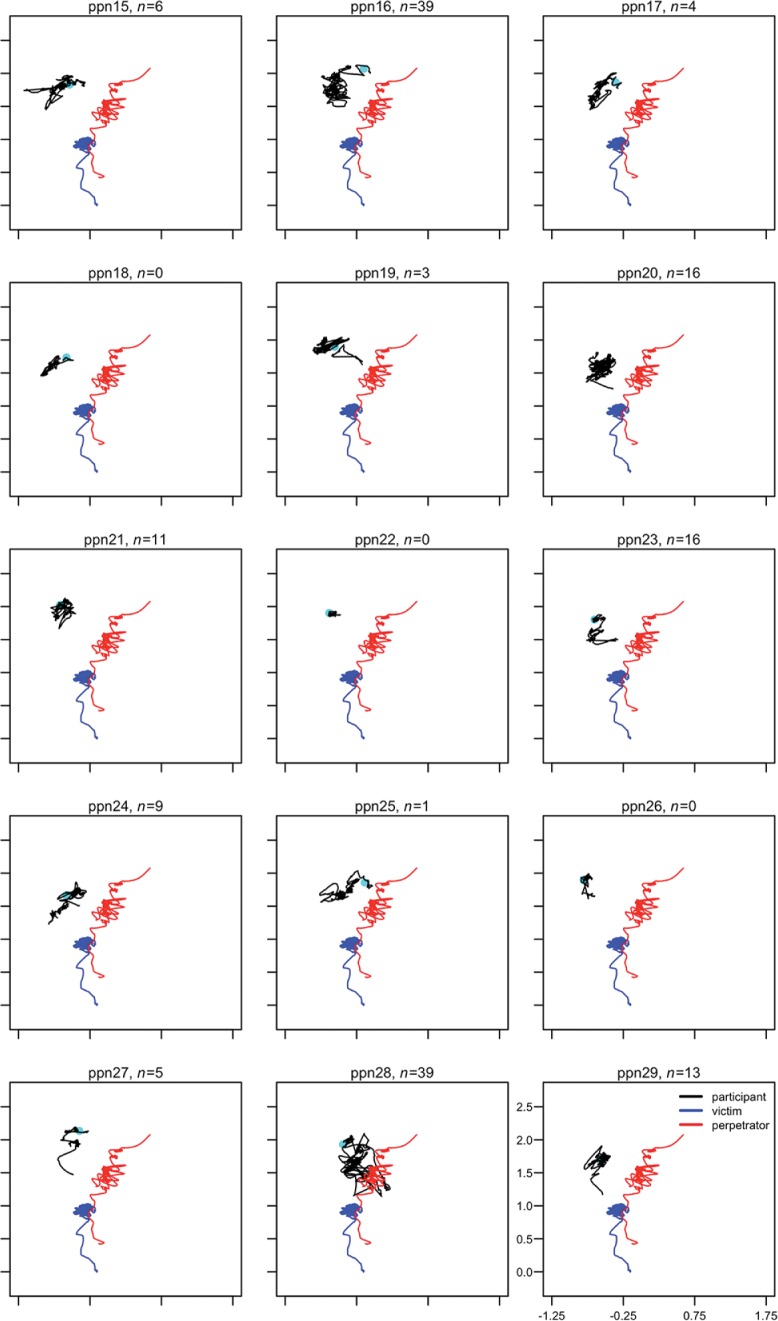
Movement of participants 15–29 and V and P during the violent conflict. *n* indicates number of interventions, cyan dots indicate the position of the participant during the conversation.

**Fig 5:**
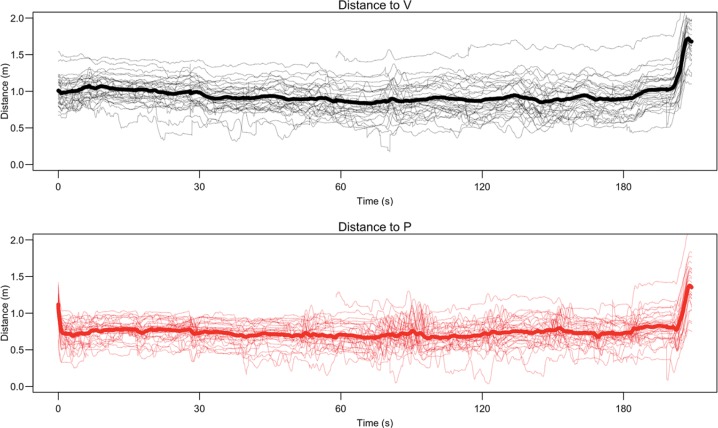
Distance to V and P during the violent conflict. The rapid increase in distance to P and V at the end is because of the physical fight. Thick lines indicate the mean distance across participants. Please note that if lines are discontinued tracking was lost (*n* = 4).

**Figs 3–**5 provide a visual representation of the movement and position of the participants with respect to V and P throughout the violent conflict. There was a significant shift in position of the participant during the conflict phase compared to the conversation phase, mean X- and Z-coordinates, *t*(28) = 2.30, *p* = .029, d = 0.43 [0.04, 0.80], mean difference = -0.06 [0.006, 0.11] and *t*(28) = 4.38, *p* < .001, d = 0.81 [0.39, 1.23], mean difference = 0.11 [0.06, 0.16] respectively. More variability in position was also observed during the conflict phase compared to the conversation phase, standard deviation of X- and Z-coordinates, *t*(28) = 2.16, *p* = .039, d = 0.40 [0.02, 0.78], mean difference = 0.036 [0.002, 0.07] and *t*(28) = 4.90, *p* < .001, d = 0.91 [0.47, 1.34], mean difference = 0.10 [0.06, 0.14] respectively. When V was attacked by P participants moved closer to V compared to the conversation phase, *t*(28) = 2.24, *p* < .03, d = 0.42 [0.03, 0.79], mean difference = 0.05 [0.004, 0.10], and the distance to V was more variable, *t*(28) = 5.79, *p* < .001, d = 1.08 [0.61, 1.53], mean difference 0.08 = [0.05, 0.10]. Mean distance to V and P during the conflict phase was 0.95 [0.87, 1.02] m and 0.74 [0.69, 0.79] m respectively. Overall, participants were most of the time in personal distance to V and P (**Table K in S1 File**). Starting position, defined as the distance of the participants during the conversation to V, was not correlated with the number of interventions, *r*_s_(29) = -.01, *p* = .955.

### Main analyses

**Fig 6:**
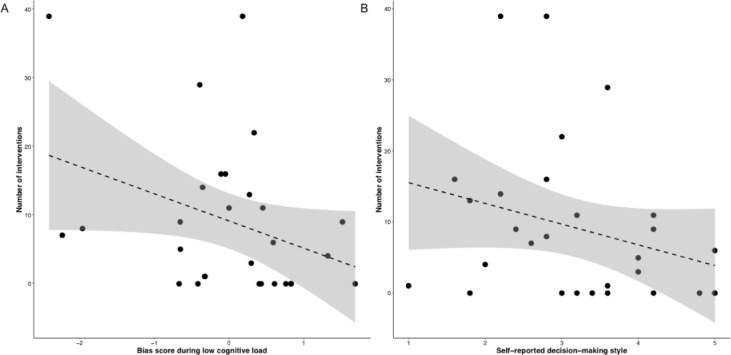
The relation between the number of interventions and behavioral reactivity to an emergency and self-reported decision-making style. Faster responses to the emergency compared to the nonemergency situation during the cued reaction time task with low cognitive load was related to more interventions during the violent conflict (A). A tendency to rate the decision to intervene as more intuitive and reflexive was related to more interventions during the violent conflict (B).

Counter to the *a priori* hypothesis, the bias score during the high cognitive load condition was not correlated to the number of interventions, *r*_*s*_(29) = -.21, *p* = .279. However, the bias score during the low cognitive load condition was correlated with the number of interventions, *r*_*s*_(29) = -.36 [-0.65, -0.003], *p* = .052 (**Fig 6A**). Participants that showed faster responses to the emergency compared to the nonemergency situation while cognition was unrestricted during the cued reaction time task, intervened more during the conflict between P and V in the virtual environment. To further quantify this effect we contrasted the intervention group (individuals with at least one physical or verbal intervention) with the no intervention group. Results showed a between group difference in bias scores in the low cognitive load condition, Mann-Whitney *U* test: *W* = 132, *p* = .049, d = 0.77 [-0.05, 1.57], but not in the high cognitive load condition, *W* = 98, *p* = .729. Under condition of limited cognitive restriction, participants who intervened had a negative bias score during the reaction time task, -0.22 [-0.70, 0.26], thus reacted faster to the emergency. The participant that did not intervene had a positive bias score, 0.50 [-0.04, 1.04], and showed slower responses to the emergency situation (**Fig A in S1 File**).

Is helping behavior related to a more intuitive decision-making process? In line with our hypotheses, we found that a tendency to rate the decision to intervene during the conflict as more reflexive was related to more interventions, *r*_*s*_(29) = -.38 [-0.66, -0.02], *p* = .042 (**Fig 6B**). Directly contrasting the intervention with the no intervention group, showed a small difference in self-reported decision-making, *W* = 129.5, *p* = .065, d = 0.80 [-0.03, 1.61]. The participants that intervened reported a more intuitive decision to intervene, 2.95 [2.47, 3.43], while the participants that refrained from intervention reported a more deliberate decision-making process, 3.78 [2.96, 4.60] (**Fig A in S1 File**). The self-reported decision-making style was not correlated with the bias scores during the cued reaction time task, *p*’s ≥ .37. Results of a linear regression analysis showed that the number of interventions during the violent conflict were predicted by the bias cores in the low cognitive load condition of the cued reaction time task, β = -0.65, *b* = -7.57 [-14.41, -.74], *p* = .032, whilst taking into account control variables (e.g., feeling of presence, previous VR experience, support for F.C. Barcelona) (**Table L in S1 File**).

### Additional analyses

**Fig 7:**
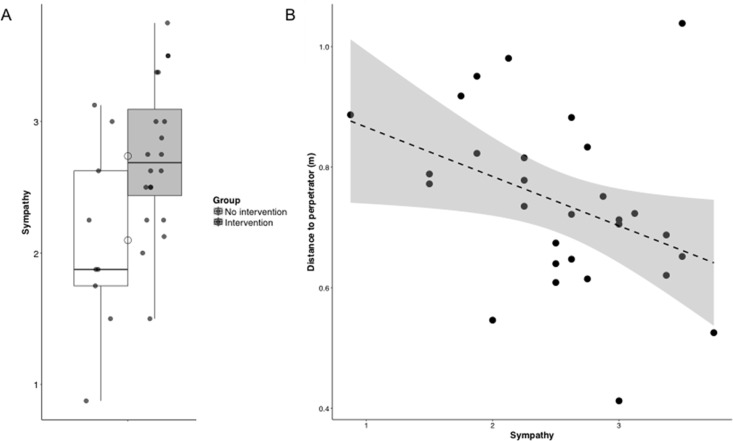
Sympathy and interventions. A disposition to experience sympathy, an other-oriented feelings during situations of distress, was higher in the intervening compared to the non-intervening participants (A), and related to a decreased distance to P during the violent conflict (B). Individual data, median and mean (circles) and the first (lower hinges) and third quartiles (upper hinges) are plotted in A.

It is likely that a mediating factor plays a role in these contrasting results. Sympathy has consistently been linked to costly helping [62–66], and recently we showed that higher trait levels of sympathy were related to faster responding to an emergency in a cued reaction time task similar to the one used here but without cognitive load manipulation [40]. Therefore, we investigated the role of a disposition to experience sympathy. Sympathy was not directly related to bias scores, low cognitive load: *r*_*s*_(29) = -.19, *p* = .34, high cognitive load: *r*_*s*_(29) = .24, *p* = .21, nor to the number of interventions, *r*_*s*_(29) = .13, *p* = .51. However, sympathy was negatively related to decision-making, *r*_*s*_(29) = -.38 [-0.65, -0.01], *p* = .044, and was higher in intervening, 2.74 [2.47, 3.01], compared to non-intervening participants, 2.10 [1.54, 2.66], *W* = 45.5, *p* = .038, d = -1.03 [-1.85, -0.19] (**Fig 7A**). In other words, a disposition to experience sympathy for others is related to a tendency to report the decision to help during a violent conflict as a consequence of an intuitive and fast process. Is trait sympathy also related more objective measures of prosociality? As the distance of the participant to a person in distress has served as a proxy for prosocial behavior [67,68], and is correlated with feelings of compassion [61], we investigated if trait levels of sympathy were associated with the distance to V and P. Results showed that sympathy was significantly related to reduced distance to P, *r*_*s*_(29) = -.44 [-0.70, -0.09], *p* = .017, but not to V, *r*_*s*_(29) = -.18, *p* = .344. Participants with a disposition to experience feelings of concern for others during distress moved closer to P during the violent conflict (**Fig 7B)**. However, these exploratory analyses are not corrected for multiple comparisons (α = .007 after Bonferroni correction) and warrant further confirmation.

## Discussion

The goal of the present study was to test whether previously measured behavioral reactivity to an observed emergency was associated with helping behavior during a later violent conflict. While acknowledging the small effects, results show the feasibility of using reactions in one emergency context to test the association with helping behavior in a different context. Reaction times during the low cognitive load condition were correlated with interventions during the violent conflict, but reaction times during the high cognitive load condition not. In addition, participants that tend to report their decision to intervene as intuitive and reflexive provided more help. Lastly, additional analyses revealed that sympathy was related to this self-reported intuitive decision-making style and to a decreased distance to the aggressor, and was higher in the intervening participants.

We did not find support for the association of helping behavior with behavioral measures under conditions of restricted cognition. While a recent meta-analysis found support for a relation between intuitive processes and prosociality [26], indicating that people are more cooperative under time pressure, this is not a causal relationship as shown by a recent registered replication report [69], see also [70]. In an intriguing study, Cowell and Decety [71] investigated the interplay between event-related potentials (ERPs) linked to automaticity and top-down control and prosocial behavior in children between three and five years of age. First, children passively observed scenes that showed either pro- or antisocial behavior of cartoon figures while simultaneously recording ERPs. Following this, children were given the opportunity to share their reward with another, anonymous, child. In contrast to a link between reflexive processes and prosocial behavior, results showed that while amplitudes of both early and late ERPs serve as a function of observed pro- and antisocial acts, only late ERPs, related to cognitive processes, were correlated with actual sharing behavior.

There is a growing body of evidence on how empathic responses, ranging from a cognitive understanding to an affective reaction, are modulated by situational and dispositional factors, but a crucial aspect is the behavior to provide help when confronted with an individual in need [72]. A wide variety of studies have provided important insight into the person-by-situation interaction [62,64,65], neural mechanisms [73,74], and neurocomputational processes [75], that contribute to the occurrence of helping behavior and functional altruism. However, this behavior is complex with several proximate causes. This explains why so far no one single trait or a combined set of traits or predictors have been found. It is likely, as also suggested by the present result, that helping behavior is the result of a complex interplay between intuitive, reflexive and deliberate, reflective mechanisms.

In an important review, Graziano and Habashi [33] suggest that there are not necessary distinguishable prosocial traits. Researchers should instead ‘think of dispositions as parts of processes and systems’, where ‘prosocial dispositions are summary terms for observed processes’ (p. 250). They suggest that the prosocial and ultimately altruistic personality is built up from thoughts (e.g., intent, beliefs), feelings (e.g., sympathy), and behaviors that are highly linked and correlated and are the result of a motivational system. The dual-process, sequential opponent motivational system [76] nicely fits recent theoretical accounts on empathy [21] and altruism [20] and provides the foundation for a wide variety of prosocial behaviors. As the name suggests two opposing evolutionary conserved motivational systems are sequentially activated when one is confronted with an emergency or other distressful event, the fight-freeze-flight and parental care system. Helping behavior is the complex interplay of these two systems that differ in terms of automaticity. The fixed action patterns of the first system are related to distress (process A) and freezing responses, and consequently inhibits helping behavior. The slower system of parental care counteracts these processes (process B) and is sympathy-driven and facilitates the occurrence of helping behavior and other forms of prosocial behavior. While the first system is thought to be automatic and reflexive, the second system is deliberative and reflective.

In line with the prediction of the motivational system as well as other studies [76,77], helping behavior was only observed after some time had passed (on average thirty seconds after onset of the conflict). The likelihood of helping increases when the second, and slower, system driven by sympathy is activated. Indeed, in line with a strong link between sympathy and prosocial behavior [64,78], dispositional levels of sympathy were higher in the intervening compared to the non-intervening participants, and sympathy was related to reduced distance to the perpetrator. Approaching the perpetrator is risky to the individual, but can be used to protect the victim, and can be seen as helping behavior by proxy. Interestingly, while the motivational system suggests that the second sympathy-related process can be described as reflective and deliberate, sympathy was related to self-reported intuitive decision-making style during the emergency in the present study. One possibility is that current popular beliefs, regardless of any empirical finding, state that heroic helping behavior is automatic and fast. ‘Doing the right thing, should require no thought’. As the statements on the decision to intervene were given after the act of helping, they might reflect this belief and/or bias. Similarly, a belief system of the individual in which helping behavior is seen as automatic, reflexive, internal might be beneficial to the actual occurrence of helping behavior [22,23,79]. Indeed, helping behavior increases when people are implicitly reminded to act without inhibition [80].

So far, the investigations of helping behavior and other prosocial tendencies have centered around a tradeoff between internal validity and ecological validity. VR provides the crucial next step in the study of these behaviors because of the combination of high experimental control and profound realism and simultaneous measurement of consistent and genuine reactions in the individual [38,55]. This technique makes it especially possible to measure phenomenological, behavioral and physiological reactions during situations that are part of everyday life, such as violent conflicts [39,81]. Here, we make use of this possibility and successfully investigate helping behavior during a violent conflict. While the combination of VR and behavioral measures, together with a strong theoretical basis, provide new vistas for the study of helping behavior, several limitations of the present study need to be considered.

First and foremost, we only tested male supporters of F.C. Barcelona and the question remains if these findings are generalizable to a more heterogeneous population. Contrary to popular belief, no consistent gender differences in helping behavior during emergency situations with [82] and without bystanders [83] have been reported so far. However, gender role [84], type of emergency [82], and group membership [39] all influence helping behavior. The later mediating factor is especially important in light of the methodological details of the present study. That is, the victim supported the same team as the participant and therefore could be considered to be part of the in-group of the participant, while the perpetrator supported a rival team and could therefore be considered to be part of an out-group. Crucially, a previous study by Slater and colleagues [39], from which the current scenario is adapted, explored these effects by directly manipulating the group membership of the victim during the violent conflict. In this study, the victim either supported the same team as the participant or not. Interestingly, not only were the number of interventions higher in the violent conflict with an in-group victim compared to the violent conflict with an out-group victim, participants interpretation of the situation changed as well. Participants confronted with an out-group victim were less involved and stated they wanted to leave the situation, and felt less confrontational than the participants that were confronted with an in-group victim. Effects of group membership and social identity have also been found in other studies on helping behavior during emergency situations [82,85–89]. While group membership or social identity are unlikely to influence the primary findings, as all the participants were F.C. Barcelona supporters and no mediating effect of level of support was found, future research should tease apart the interaction between reflexive and reflective reactions, group membership and social identity and helping behavior.

Another consideration is the measurement of helping behavior. While we were able to measure helping behavior in a high impact situation with high personal relevance, the behavior was measured while the participant was in a virtual world. This raises the question how much of the observed behavior and effect is generalizable across situations and worlds, that is from the virtual to the real world. Indirect evidence suggests it is. A short VR experience influences real-life helping behavior measured at a later time [90], and a recent study found that VR-induced changes in implicit racial biases are stable over a short period of time [91]. Lastly, it is important to note that the observed effects were small in nature. While the cued reaction time task [40], VR scenario [39], and questionnaires all have successfully been used and validated, future studies should directly or conceptually replicate the present study with a larger and more diverse sample size, as well as with multiple measures of inter-individual differences in reflexive- and reflective processes.

In conclusion, we have used a multilevel approach and incorporated behavioral reactivity, self-reported decision-making, and proxemics during Virtual Reality to study helping behavior during a violent conflict. Results showed that faster responses to an emergency situation while cognition is not restricted are associated with later helping behavior and suggest an important role for a disposition to experience other-oriented responses to distress in the occurrence of helping behavior.

## Supporting information

S1 File Supporting information. (Table A) Sample characteristics, (Table B) Decision-making questionnaire items and descriptive statistics, (Table C) Interpersonal reactivity index scales, descriptive statistics and comparison with previous samples, (Table D) Descriptive statistics for the cued reaction time task, (Table E) Place illusion questionnaire items and descriptive statistics, (Table F) Plausibility questionnaire items and descriptive statistics, (Table G) Interview responses for feelings/responses during the conflict, (Table H) Interview responses for realism of responses, (Table I) Interview responses for aspects that would lead to a higher likelihood of intervention, (Table J) Interview responses for factors leading to feelings of being outside of the situation, (Table K) Time spend in proximity of the victim and perpetrator, (Fig A) Between group differences in behavioral reactivity to an emergency and self-reported decision-making style during the violent conflict, (Table L) Outcome of the regression analysis.
(DOCX)

S1 Data A comma-separated values (CSV) file containing the data. (CSV)
