# Supplementary material for: The relation between bystanders’ behavioral reactivity to distress and later helping behavior during a violent conflict in virtual reality
Source: PLoS One. 2018 Apr 19;13(4):e0196074. doi: 10.1371/journal.pone.0196074 (PMC5908166; doi:10.1371/journal.pone.0196074)
Supplement: S1 File — (Table A) Sample characteristics, (Table B) Decision-making questionnaire items and descriptive statistics, (Table C) Interpersonal reactivity index scales, descriptive statistics and comparison with previous samples, (Table D) Descriptive statistics for the cued reaction time task, (Table E) Place illusion questionnaire items and descriptive statistics, (Table F) Plausibility questionnaire items and descriptive statistics, (Table G) Interview responses for feelings/responses during the conflict, (Table H) Interview responses for realism of responses, (Table I) Interview responses for aspects that would lead to a higher likelihood of intervention, (Table J) Interview responses for factors leading to feelings of being outside of the situation, (Table K) Time spend in proximity of the victim and perpetrator, (Fig A) Between group differences in behavioral reactivity to an emergency and self-reported decision-making style during the violent conflict, (Table L) Outcome of the regression analysis. (DOCX) [file pone.0196074.s001.docx]

| **Table A. Sample characteristics** |  |
| --- | --- |
| *Question* | *Median (IQR)* |
| General interest in football *^1^* *^(not at all) to 7 (very much)^* | 5 (4) |
| Support for F.C. Barcelona *^1^* *^(not at all) to 7 (very much)^* | 5 (2) |
| Experience in Virtual Reality *^1^* *^(never) to 7 (many times)^* | 2 (3.5) |
| Video games played per week *^1 (0 hours) to 7 (>9 hours)^* | 1 (3) |
| Informatics expertise *^1 (beginner) to 7 (expert)^* | 4 (1.5) |
| Programming expertise *^1 (beginner) to 7 (expert)^* | 1 (1) |
| IQR = interquartile range. | |

| **Table B. Decision-making Questionnaire** |  |
| --- | --- |
| *Question* | *Median (IQR)* |
| I tried to intervene, because my reaction was | 3 (3) |
| When the guy with the Real Madrid shirt started to insult the other guy, my verbal intervention was: | 4 (3) |
| When he started to insult the other guy, my physical intervention was: | 3 (3) |
| When the guy with the Real Madrid shirt started to push the other guy, my verbal intervention was: | 4 (3) |
| When he started to push the other guy, my physical intervention was: | 3 (2) |
| *Overall decision-making* | 3.2 (1.8) |
| The scale ranged from 1 (intuitive/fast) to 5 (reasoned/slow). IQR = interquartile range. If the participants did not intervene, the question referred to the decision not to intervene. | |

| **Table C. Interpersonal Reactivity Index** | | | |
| --- | --- | --- | --- |
| *Question* | *Present study* | *Pérez-Albéniz et al. study 2* | *Pérez-Albéniz et al. study 3* |
| Sympathy (empathic concern) | 2.54 ± 0.68 | 3.88 ± 0.58 | 3.65 ± 0.59 |
| Personal distress | 1.40 ± 0.80 | 2.59 ± 0.68 | 2.62 ± 0.68 |
| Perspective taking | 2.50 ± 0.63 | 3.33 ± 0.69 | 3.35 ± 0.68 |
| Fantasy | 2.37 ± 0.70 | 3.26 ± 0.79 | 3.15 ± 0.79 |
| Mean score ± standard deviations reported for the present study and the scores for the male participants from study 2 (*n* = 1997) and 3 (*n* = 515) from Pérez-Albéniz et al. 2003. | | | |

| **Table D. Mean reaction times ± standard deviations for the cued reaction time task** | | |
| --- | --- | --- |
|  | Low cognitive load | High cognitive load |
| Emergency | 317.31 ± 38.67 | 331.62 ± 48.62 |
| Nonemergency | 318.86 ± 44.11 | 329.93 ± 44.75 |
| Emergency – Nonemergency bias score | -1.55 ± 25.70 | 1.69 ± 22.53 |
|  |  |  |

| **Table E. Place illusion Questionnaire** |  |
| --- | --- |
| *Question* | *Median (IQR)* |
| Please rate your sense of being in the discussion in the bar, on the following scale from 1 to 7, were 7 represents your normal experience of being in a place | 5 (3) |
| To what extent were there times during the experience when the discussion in the bar was the reality for you? | 4 (2) |
| When you think back about your experience, do you think of the situation in the bar more as images that you saw, or more as somewhere you visited? | 5 (3) |
| During the time of the experience, which was strongest on the whole, your sense of being in the bar, or of being in the real world of the laboratory? | 5 (3) |
| *Overall Place illusion* | 4.25 (2.63) |
| The scale ranged from 1 (low presence) to 7 (high presence). IQR = interquartile range |  |

| **Table F. Plausibility Questionnaire** |  |
| --- | --- |
| *Question* | *Median (IQR)* |
| How much did you behave during the discussion in the bar as if the situation were real? | 4 (3) |
| How much was your emotional response during the discussion the same as if it had been a real situation? | 3 (4) |
| How much were your thoughts you had during the discussion the same as if it had been a real situation? | 4 (4) |
| To what extent were your physical responses the same as if it had been a real situation? | 3 (2) |
| How much did you behave as if the guys were real? | 3 (3) |
| How much was your emotional response to the two guys as if they were real? | 5 (3) |
| How much were your thoughts in relation to the two guys as if they were real? | 4 (4) |
| How much were you thinking things like “I know these guys are not real” but then surprisingly finding yourself behaving as if they were real? | 4 (3) |
| How much did you have physical responses to the guys as if they were real? | 3 (3) |
| *Overall Plausibility* | 3.67 (2.17) |
| The scale ranged from 1 (low presence) to 7 (high presence). IQR = interquartile range |  |

| **Table G. What feelings/responses did you have during the argument?** | |
| --- | --- |
| *Code* | *Percentage of responses* |
| Anger | 22.64 |
| Helplessness | 15.09 |
| Sympathy | 9.43 |
| Need to help | 7.55 |
| Distress | 7.55 |
| Rational | 5.66 |
| No feelings or indifference | 5.66 |
| Not real | 5.66 |
| Surprised | 3.77 |
| Unfairness | 3.77 |
| Fear or avoidance | 3.77 |
| Implicated | 3.77 |
| Observing | 3.77 |
| Pride | 1.89 |
| *Total number of statements* | *53* |
|  |  |
|  | |

| **Table H. Were your responses realistic?** | | |
| --- | --- | --- |
| *Code* | *Percentage of responses* |  |
| Realistic or quite realistic | 57.89 |  |
| Lacked interaction | 15.79 |  |
| Contrasts VR and reality | 13.16 |  |
| Detached | 13.16 |  |
| *Total number of statements* | *38* |  |
|  | | |

| **Table I. What would have made you more likely to intervene?** | | |
| --- | --- | --- |
| *Code* | *Percentage of responses* |  |
| More interactively | 38.89 |  |
| More realism | 22.22 |  |
| More aggression | 22.22 |  |
| More rapport | 13.89 |  |
| More bystanders | 2.78 |  |
| *Total number of statements* | *36* |  |
|  | | |

| **Table J. What factors made you feel outside of the situation?** | | |
| --- | --- | --- |
| *Code* | *Percentage of responses* |  |
| It is a VR experiment | 16.33 |  |
| No response from characters during the argument | 12.24 |  |
| Own emotions or thoughts | 12.24 |  |
| Lack of sense of touch | 10.20 |  |
| No attention of P to participant | 10.20 |  |
| Not realistic | 10.20 |  |
| Technical issues (POV, clipping, size proportions, tracking) | 8.16 |  |
| Dialogue not realistic | 6.12 |  |
| It looked like a game or movie | 6.12 |  |
| Unknown V and P | 4.08 |  |
| Content of conflict | 4.08 |  |
| *Total number of statements* | *49* |  |
|  | | |

| **Table K. Mean ± standard deviations of the time spend in proximity of V and P.** | | | | |
| --- | --- | --- | --- | --- |
|  | Public | Social | Personal | Intimate |
| V – Conversation | - | 14.19 ±32.65 | 84.39±37.29 | 0.08±0.43 |
| V – Conflict | - | 17.37±32.13 | 114.47±33.58 | 1.55±6.86 |
| P – Conflict | - | 2.03±4.43 | 123.73±18.08 | 7.62±17.59 |
| The definition for social distances from Hall (1966) was used. Time in seconds is reported. V = victim, P = Perpetrator | | | | |


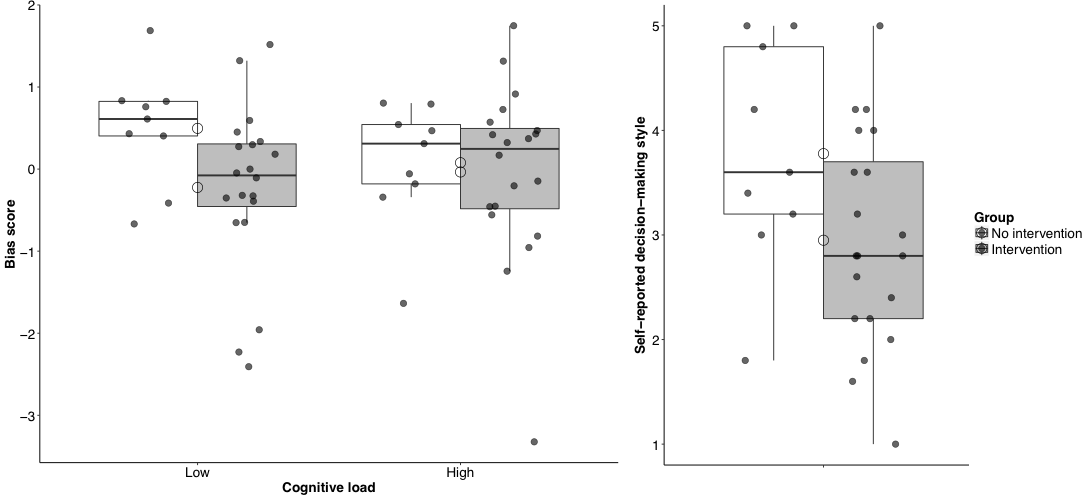


**Figure A. Between group differences in behavioral reactivity to an emergency and self-reported decision-making style during the violent conflict.** Participants that intervened compared to participants that did not intervene showed faster responses to an emergency compared to the nonemergency situation during the cued reaction time task with low cognitive load (right) and reported the decision to act as intuitive and fast (left). Individual data, median and mean (circles) and the first (lower hinges) and third quartiles (upper hinges) are plotted.

| **Table L. Outcome of the regression analysis for the number of interventions during the violent conflict** | | | |
| --- | --- | --- | --- |
| *Question* | *b* | β | *p* |
| *Step 1: Control variables* | | | |
| Overall model: *F*(10, 26) = .43, *p* = .912, R^2^ = .21 | | | |
| Constant | -.36 [-43.71, 42.99] |  | .986 |
| General interest in football | 2.12 [-5.18, 9.42] | .35 | .548 |
| Support for F.C. Barcelona | -1.16 [-7.49, 5.17] | -.19 | .703 |
| Experience in Virtual Reality | .72 [-3.11, 4.55] | .11 | .696 |
| Perspective taking | .02 [-10.88, 10.92] | .001 | .996 |
| Personal distress | -.84 [-7.84, 6.16] | -.06 | .803 |
| Fantasy | -.24 [-11.44, 10.97] | -.02 | .965 |
| Sympathy (empathic concern) | -.71 [-10.65, 9.22] | -.04 | .881 |
| Place Illusion | -3.16 [-9.51, 3.20] | -.38 | .308 |
| Plausibility | 4.52 [-2.14, 11.18] | .48 | .169 |
| Starting position | 2.90 [-26.43, 32.23] | .05 | .837 |
| *Step 2: Predictor variables* | | | |
| Overall model: *F*(13, 26) = 1.01, *p* = .496, R^2^ = .50 (R^2^ change = .29, *p*  = .10) | | | |
| Bias score during low cognitive load | -7.57 [-14.41, -0.74] | -.65 | .032 |
| Bias score during high cognitive load | -2.38 [-7.89, 3.14] | -.21 | .369 |
| Decision-making style | -2.56 [-8.90, 3.78] | -.24 | .398 |
| In step 1 the control variables are entered, while in step 2 the predictor variables are entered. *b* = unstandardized coefficients [lower and upper bounds of the 95% confidence interval], β = standardized coefficient. | | | |
